# Stromal Androgen Receptor in Prostate Cancer Development and Progression

**DOI:** 10.3390/cancers9010010

**Published:** 2017-01-22

**Authors:** Damien A. Leach, Grant Buchanan

**Affiliations:** 1 The Basil Hetzel Institute for Translational Health Research, The University of Adelaide, Adelaide 5011, Australia; Grant.Buchanan@act.gov.au; 2 Department of Surgery and Cancer, Imperial College London, Hammersmith Hospital Campus, Du Cane Road, London W12 0NN, UK; 3 Department of Radiation Oncology, Canberra Teaching Hospital, Canberra 2605, Australia

**Keywords:** prostate cancer, stroma, fibroblasts, androgen, androgen receptor

## Abstract

Prostate cancer development and progression is the result of complex interactions between epithelia cells and fibroblasts/myofibroblasts, in a series of dynamic process amenable to regulation by hormones. Whilst androgen action through the androgen receptor (AR) is a well-established component of prostate cancer biology, it has been becoming increasingly apparent that changes in AR signalling in the surrounding stroma can dramatically influence tumour cell behavior. This is reflected in the consistent finding of a strong association between stromal AR expression and patient outcomes. In this review, we explore the relationship between AR signalling in fibroblasts/myofibroblasts and prostate cancer cells in the primary site, and detail the known functions, actions, and mechanisms of fibroblast AR signaling. We conclude with an evidence-based summary of how androgen action in stroma dramatically influences disease progression.

## 1. Introduction

Histological assessment of solid tumours has been used in combination with clinical parameters for many decades to inform both diagnosis and management decisions. In the emerging era of immunotherapeutics and personalized medicine, histology and molecular assessment is playing an increasingly important role in defining prognosis and individualised treatment options. Assessment now often includes protein activity and mutation status in addition to extent and level within a tumour sample, as well as markers of tumour activity, mitosis and turnover. For breast cancer, levels and extent of oestrogen receptor (ER), progesterone receptor (PR, as a marker of ER function) and HER2 are used to broadly categorize a tumour and inform on the benefit of anti-estrogen agents (e.g., tamoxifen) or tyrosine kinase inhibitors. Similarly, assessment of colon cancer includes EGRF, KNAS and UHA1; of melanoma, BRAF; of lung cancer, EGRF, ALK, KRAS and ROS-1; and of leukaemia a panel of markers for typing. Prostate cancer remains an anomaly in this regard. Despite being the most common, non-skin, cancer, and the leading cause of cancer related death, prognosis and treatment is generally defined using clinical and pathological parameters established decades ago. The predominant histological patterns of glandular disorganization are captured in the Gleason score, which together with clinical assessment and/or medical imaging regarding the extent of disease within the prostate and any extracapsular disease, are combined to provide prognostic information. Serum prostate specific antigen (PSA) testing was introduced over 20 years ago, and although useful in stratification of patients for investigation, risk of recurrence following definitive treatment and disease monitoring, is not a particularly useful in a prognostic sense. Intriguingly, the lack of prognostic markers available to patients and clinicians is predicted to have led to both over and under treatment of patients, with financial and social implications for both patients and the health care system. Currently, no histological markers are routinely used to determine prostate cancer prognosis, or inform on the usefulness of androgen ablation strategies. A key limitation in this regard is the multi-focal nature of most prostate cancers, and the inherent heterogeneity within cancerous epithelia of individual patients. One alternative being explored is the assessment of reactive changes occurring within the surrounding stroma.

Despite being generally regarded as a simple supportive structure for the specialised cells within an organ, the stroma is actually vital to organ development and homeostasis, and plays a significant role in both carcinogenesis and metastasis. The stroma is composed of a mixture of smooth muscle cells, fibroblasts, immune cells, lymphatics, vasculature and extracellular matrix (ECM) as well as via a rich array of secreted factors, hormones, enzymes and other soluble second messengers. Along with direct cell-cell interaction, these factors mediate communication between stromal constituents and bidirectional signalling between stromal and epithelial compartments, which is observed in all organs and is vital for normal development. With carcinogenesis and with tumour growth, substantial changes are found in stromal constituents and behaviour. Cancer stroma is characterised by a loss of smooth muscle cells and a predominance of activated myofibroblasts, termed cancer associated fibroblasts (CAFs), that enable carcinogenesis, stimulate tumour growth and contribute to invasion [1]. The CAFs which surround the cancerous gland development from multiple sources, circulating marrow derived progenitors, adipose tissue, and fibroblasts from distant organs, but a vast majority are reported to develop from the resident fibroblast population [2,3]. Indeed, the extent of transformation of the fibroblasts can associate with disease progression, potentially through providing paracrine cues to disrupt and disaffect homeostasis. The prostate provides a compelling example of intra-compartmental signalling that influences normal development and malignant cell behaviour. The growing appreciation of the role played by prostate stroma in carcinogenesis, tumour behaviour and response to conventional therapy is driving new innovation in research and treatment.

Prostate cancer remains the most commonly diagnosed non-skin malignancy and second leading cause of cancer related death in US men, with invasion and metastasis from the primary site reducing patient survival by 50%. Current clinical nomograms utilize imaging, clinico-pathological parameters and serum leak of epithelial produced PSA to broadly stratify cancers according to risk of progression following treatment, but cannot accurately predict tumour progression at the time of diagnosis, or the timeframe in which progression might be clinically significant. As a consequence, it is believed that many patients either incur treatments and their associated side effects unnecessarily, or are not receiving the appropriate therapy or monitoring for aggressive disease.

Androgens are a key factor in prostatic development, homeostasis and malignancy. With respect to the former, early in vivo studies showed that the absence of hormone responsive stroma prevented epithelial cell differentiation and organ and glandular development [4,5]. Nonetheless, the vast majority of androgen and androgen receptor (AR) research has been focussed on epithelial cancer cells because of the response of these cells, and prostate tumours, to androgen deprivation. The purpose of this review is to provide an emerging review of hormone signalling in the fibroblasts and myofibroblasts of the prostate (the most prominent stromal cells in prostate cancer) and how it controls stromal-epithelial interactions in the primary tumour setting, and to describe how changes in this pathway are emerging as a key determinants of prostate cancer progression and outcome.

## 2. Stromal AR in Prostate Cancer Outcome

Continued growth of metastatic prostate cancer cells during complete androgen blockade, in both clinical and experimental settings, is the result of mechanisms permissive for continued function of AR and/or those of its activated pathways despite combined AR/androgen targeting. Although increased AR expression in the epithelial cancer cells is one such mechanism, there is inconsistent evidence that it contributes to development or progression of the primary tumour. As reviewed in Tamburrino et al. [6], epithelial AR levels in primary prostate cancers has been inconsistently related to patient outcome, with 20% of studies suggesting high cancer AR as a prognostic marker of good outcome, 26% showing high AR as a prognostic marker of poor outcome, and the majority showing no relationship (Table 1). In comparison, for the smaller number of studies looking at stroma, a loss of stromal AR has universally been related to the cancerous state, high risk clinical parameters, disease progression and/or poor outcome (Table 2). In these studies, the term stroma refers to the cells directly adjacent to the epithelial or cancerous cells, which are usually noted for their fibroblast appearance. In a study of twenty patients, Mohler et al., showed lower intensity immunostaining of AR in cancer stroma compared to regions of benign prostatic hyperplasia [7], but there was no correlation with cancer progression, possibly due to the small cohort size. However, in larger studies, statistically significant associates were made. In four studies, in cohorts of 53 patients (radical prostatectomy (RP) samples), 152 patients (two separate cohorts, 78 transurethral resection of the prostate (TURP), and 74 biopsy), 96 patients (RP), and 53 patients (RP), low stromal AP was significantly associated with biochemical relapse and response to castration [8,9,10,11]. Other clinical parameters were also associated, including Gleason score and disease stage. We have shown in a cohort of 64 patients that low stromal AR expression inversely associates with patient outcome, to which we later added that the using FKBP5 as a marker of AR activity could be combined with AR levels to for an even stronger inverse relationship with patient outcome [12,13]. Importantly, this cohort had benign and cancers samples taken from each patient, which showed that the loss of AR was specific to the cancer associated stroma. Overall, all currently published patient-based studies indicate that lower AR in prostate cancer stroma is associated with disease progression and/or worse outcome, implying that stromal AR is protective. It will be important to know if this has prognostic significance, both in terms of patients most at risk of developing advanced disease and the potential response of an individual tumour to androgen deprivation. These findings are distinct from the potential beneficial effects of stromal AR in preventing caner initiation and development, which is discussed further below.

## 3. Androgen Signalling

**Figure 1:**
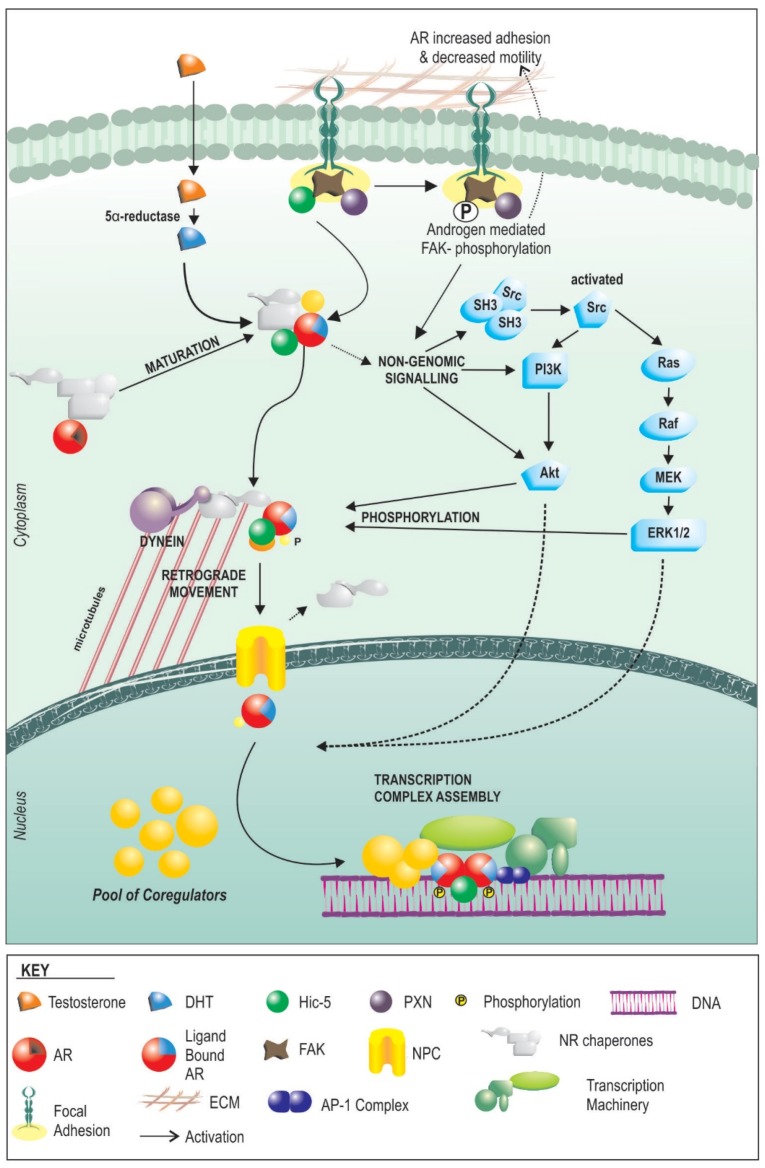
Schematic of androgen receptor (AR) signalling in fibroblasts/myofibroblasts. Serum testosterone enters the cell, converts, via the 5α-reductase enzyme, into dihydrotestosterone (DHT). This then binds to the AR which resides in the cytoplasm, bound to chaperones, causing a conformational change and activation of the AR. The AR can then cause a series of non-genomic effects via kinase pathways, but also shuttles via microtubules to the nucleus which it enters via nuclear pore complexes (NPC). Concomitantly, activated AR also causes nuclear translocation of focal adhesion proteins such as Hic-5 (thus altering adhesiveness and movement of cells) which it uses as a co-regulator, along with a pool of cofactors and other co-regulators (some of which are fibroblast/stroma specific) to combine with transcriptional machinery and regulate gene expression.

Androgens act primarily through their cognate receptor, the androgen receptor (AR), which is a potent transcription factor with broad tissue distribution and a major mediator of cellular function and homeostasis. Androgens are vital for growth and maturation of the prostate. However, the mechanism, regulation, and outcomes of AR signalling are based primarily on whole body physiological responses, and molecular studies in predominantly cancerous epithelial cells. AR signalling (Figure 1), in most basic terms this starts with cellular internalization of circulating androgens such as testosterone (T). Androgens then bind directly to the AR with variable affinity, or in the case of T may be first metabolized to the more potent dihydrotestosterone (DHT) via the enzyme 5-alpha reductase. Steroid binding to the AR occurs in the cytoplasm, where the receptor resides in an inactive state in complex with molecular chaperones, such as HSP90, and other proteins. Binding and activation in the initiation of genomic signalling pathways including PI3K-AKT, and ERK. Activation of AR also results in alteration sin the interaction with chaperones, allowing for translocation to the nucleus via movement along microtubules. Nuclearisation culminates in the interaction of the AR with chromatin, and ultimately regulation of the cellular transcriptional profile. The transcriptional response to androgens is modulated by the availability of steroid and the cellular complement of pioneer, coregulatory and chaperone proteins.

## 4. How AR Signaling in the Stroma Works

Despite observations of AR in the stroma being important in all stages of prostate development and carcinogenesis, until recently little was known about the mechanics of AR function in that cellular compartment. In the benign prostate the predominant stromal cells are smooth muscle cells, a majority of which strongly express AR. Myofibroblasts are the predominant cell type present in the tumour stroma, and although they can be seen to express AR and show physiological and molecular responses to androgens in vivo [12], primary human fibroblasts shed AR expression within 1–2 passages in culture. To overcome this limitation, two engineered human prostate myofibroblast cell lines have been developed, WPMY-1 and PShTert-ARs [41,42]. Of these two, only PShTert-AR cells stably express AR, which has a similar AR binding patterns and gene regulation to primary and in vivo mesenchyme [12,43], as well as being able to inhibit fibroblast proliferation replicative of in vivo studies of human prostate, as well as being able to excite epithelial cells proliferation just as mesenchyme in mouse recombination studies [12]. Furthermore, androgen action in these myofibroblast cells lines validates in patient NPF and CAFs [12].

In general terms, the molecular action of AR function in fibroblast lineage cells appears to follow the same general basic principles as AR in epithelial cells, but with some key differences that radically alter the cellular response (Figure 1). At the front end, HSP90 appears to be equally essential for AR function in both cell types [44], and the receptor traffics to the nucleus only following steroid binding [45]. Importantly however, when we recently compared the global transcriptional response to androgens, only around 10% of genes regulated by androgens in prostate myofibroblasts were common with those regulated in epithelial cells [12]. This appears to be the result of lineage-specific differences in the expression of co-regulators and pioneer factors. Cofactors are a diverse set of proteins that exert their effects on AR by influencing stability, ligand binding, interaction with other proteins, DNA interactions via modification to histone acetylation, methylation and sumoylation, recruitment of the transcriptional machinery or baseline activity. The expression and ratio of co-regulators are different between epithelial cells and non-epithelial cells of the prostate [46]. As an example, we have shown that the mesenchymal specific co-regulator, Hic-5 affects regulation of over 50% of genes targeted by androgen receptor in fibroblasts [45]. Pioneer factors are proteins that regulate targeting and/or activity of transcription factors to specific regions of DNA. Unlike epithelial cancer cells that utilize the forkhead protein, FOXA1 as the primary AR pioneer factor [47,48,49,50], we have shown that prostate fibroblasts appear to use the AP1 complex, and JUN in particular, leads to regulation of distinct molecular pathways in fibroblasts [43]. As one example, JUN driven fibroblast specific regulation of licensing factor FBXO32 by AR results in a switch to inhibiting of fibroblast proliferation by androgens.

## 5. Stromal AR in Prostate Development

**Figure 2:**
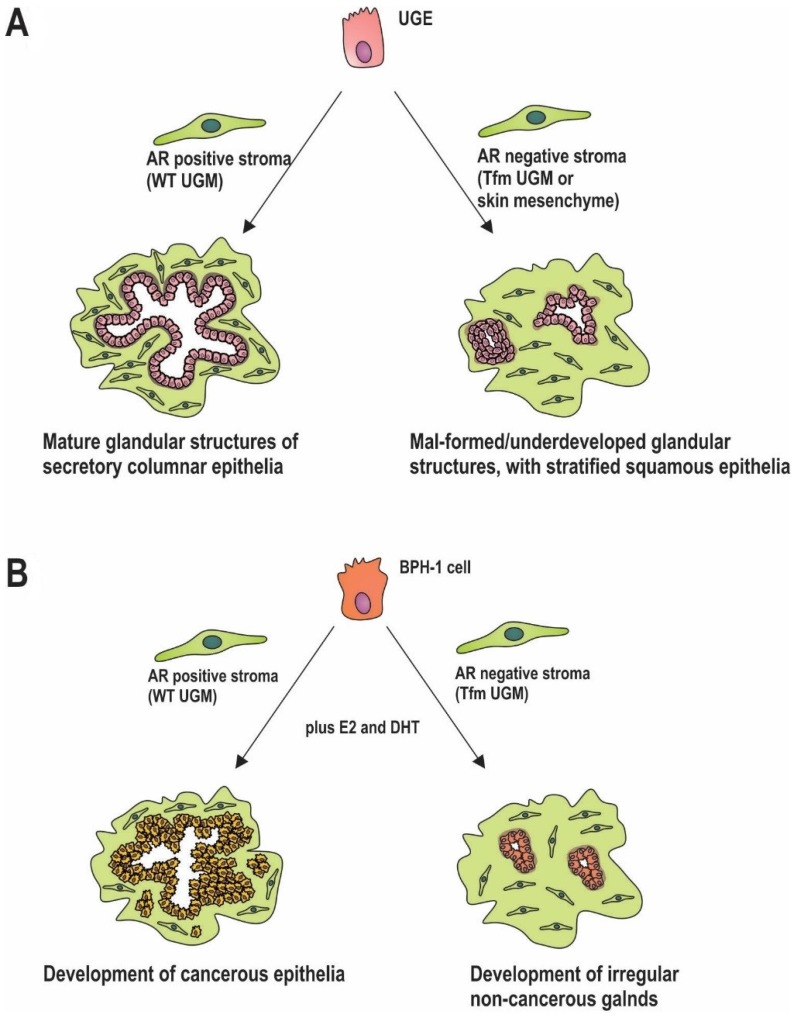
Impact of AR expression on prostate development and carcinogenesis. (**A**) Stromal AR is required for prostate development. In mouse models combining embryonic urogenital epithelia (UGE) with AR positive urogenital mesenchyme (UGM) results in normal epithelial structures, which doesn’t occur when UGE is combined with AR negative or non-functionally AR containing mesenchyme; (**B**) AR is needed in stroma for cancer initiation. When transformed BPH-1 cells are grown in mice in the presence of AR positive mesenchyme cancer initiation and development can occur, but when combined with AR negative stroma, only small, irregular, non-cancerous glands form.

In the embryonic/developing prostate the urogenital mesenchyme (UGM) is comprised of AR positive precursors to fibroblast and smooth muscle cells, similar to myofibroblasts [51,52,53]. Supporting a role for stromal androgen signalling throughout prostate development, expression of the AR occurs higher and earlier in this compartment than in epithelia, and is maintained throughout maturation. This has been demonstrated in tissue recombination models, where AR positive UGM leads to normal growth and glandular differentiation of urogenital epithelia (UGE). In contrast, AR negative mesenchyme from skin results in differentiation of UGE to stratified squamous epithelia [4,54] (Figure 2A). Studies utilizing cells extracted from testicular feminized (Tfm) mice, which have a non-functional AR, further clarify the importance of stromal androgen signalling. When wild type (WT) UGM is combined with UGE from Tfm mice, prostatic structures develop normally. In contrast, tissues generated from Tfm UGM and either WT UGE or Tfm UGE fail to generate glandular architecture [55] (Figure 2A). Additional studies demonstrate poor differentiation of prostatic ducts and glandular acini in mice that lack stromal AR [56] (Figure 2A). Although androgen signalling in the mature prostate epithelia is primarily responsible for secretion of seminal fluid constituents, including prostate specific antigen (PSA) [57], this process can also be modulated by the prostatic stroma [58,59]. In the mature prostate, AR positive smooth muscle cells are the predominant cell type. In vitro, AR action in fibroblasts increases epithelial AR activity, as measured by in vitro assays of AR activity [60], and results in increased in epithelial PSA production [61]. Collectively, these findings implicate stromal AR activity in development, maintenance and biological function of adjacent epithelia. More broadly, there appears to be a universal role for mesenchymal hormone signalling in the development of both male and female reproductive organs, with expression of the appropriate hormone receptors in adjacent stroma critical for subsequent organ-specific responses to oestrogen, progesterone, and testosterone [62,63,64,65].

## 6. Stromal AR in Carcinogenesis

The role of stromal androgen signalling in prostate carcinogenesis is becoming more and more prominent [66,67,68]. Stromal AR activity is also required for tumour formation in prostatic epithelia in recombinant mouse models [69]. AR negative initiated epithelial cells were implanted into castrate mice flanks along with AR negative or positive UGM. Mice were then treated with or without androgen and estrogen. In mice implanted with epithelia alone, there was no tumour formation under any treatment condition. Where mice were implanted with initiated epithelium and AR positive UGM, tumour formation occurred in 36% (*n* = 30/84) of hormone treated mice but <0.5% (*n* = 1/218) of untreated mice [69]. Whilst that study did not specifically compare AR positive versus AR negative UGM, it did demonstrated the importance of stroma in early stage cancer, and the potential role of stromal AR signalling in tumour formation. A role in early transformation was addressed more recently by implantation of initiated prostate epithelia (via knockdown of tumour suppressors PTEN and p53) with wild-type or Tfm mesenchyme [70]. When initiated, epithelia were combined with WT mesenchyme, tumour formation occurred following hormonal stimulation (Figure 2B). In contrast, when combined with the AR negative Tfm mesenchyme, the result was merely the development of small non-invasive growths (Figure 2B). Significantly, the presence of AR in the epithelial cells did not affect those processes [70]. Similarly, the spontaneous development of prostatic intraepithelial neoplasia seen in PTEN+/− mice, was decreased in offspring bred with stromal AR knockout mice (ARKO) [71]. Furthermore, inhibiting the AR chaperone, HSP90, in CAFs, thereby reducing the AR activity, retards growth of patient derived cancer cell and CAF recombinant xenografts in mice [44].

AR positive stroma is also capable of inducing prostate tumour formation from grafted AR negative benign prostatic hyperplasia (BPH)-1 cells [69], but is hindered in mice which lack stromal AR in comparison to stromal AR positive mice [72]. Perhaps significantly, in men of African descent where there is a higher incidence of prostate cancer compared to Caucasian men, there is reportedly higher stromal AR expression [73]. Regardless, the evidence collectively supports stromal AR signalling acting to induce prostate cancer cell proliferation and potentially play an important role in early prostate carcinogenesis. Thus, it would appear that stromal AR plays an important and often overlooked role in early prostate carcinogenesis. It is important, however, to distinguish this from the potential role of decreased stromal AR in cancer progression and metastasis (see Section 2).

## 7. Why Is Stromal AR Lost?

Despite the relationship between clinical outcome and stromal AR loss highlighted in Table 2, the mechanisms underpinning altered AR expression in this compartment in some, or perhaps all, prostate tumours are unknown. One hypothesis is AR negative/low CAFs represent a subgroup of an initial CAF population that undergoes clonal selection in some manner. We have previously reported that AR action in myofibroblasts inhibits their intrinsic proliferation [12], which might provide a selective pressure for the AR negative/low CAF population over those that highly express the receptor. A second tier question is how variable AR expression occurs in stroma in the first place. Cellular variability in ligand availability is one possibility. We know that AR signalling in stroma is less sensitive than in epithelial cells, and thus more vulnerable to systemic changes in androgen levels, or on altered supply based on local tumour microarchitecture and/or vascular supply. Decreased ligand availability will manifest as decreased AR stabilization and increased receptor turnover. An alternative and relatively unexplored possibility is that of stromal mutagenesis occurring distinct from genetic alterations within the cancer cells themselves. Some studies using mixed prostate tumour samples have, for example, paradoxically identified inactivating AR mutations that have been difficult to rationale in the context of almost invariable AR driven epithelial disease [74]. It is tempting to speculate that some of those mutations may have been captured from stromal components. Epigenetic regulation could also be involved, as changes in methylation state are known to regulate AR expression [75]. Alternatively, p53 has been show to negatively affect AR interactions leading to receptor stabilization and activity [76], and forms part of a stromal signature in prostate cancer associated with biochemical relapse [77]. However, down regulated genes weren’t assessed as part of that study, so it is currently unclear if there is a direct relationship.

There is a clear need for a more contemporary analysis of cancer cells associated with high and low stromal AR content, and to track mutational and transcription events within each compartment. It is likely that events in one or both compartments of a tumour will can change the way cancer cells interact with their microenvironment. Paracrine factors such as interleukins, interferons, and miRNAs have all been reported to reduce AR levels [78,79,80,81,82]. Nitric oxide is a product of certain events within cancer cells, inhibits AR expression and activity, and plays a role in cancer progression and metastasis [83,84,85]. Given the potential prognostic importance of stromal AR expression, studies need to extend beyond speculative hypotheses to address in real time how AR levels fluctuate within a tumour sample.

## 8. Possible Mechanisms for the Involvement of Stromal AR Signalling in Cancer Progression and Outcome

**Figure 3:**
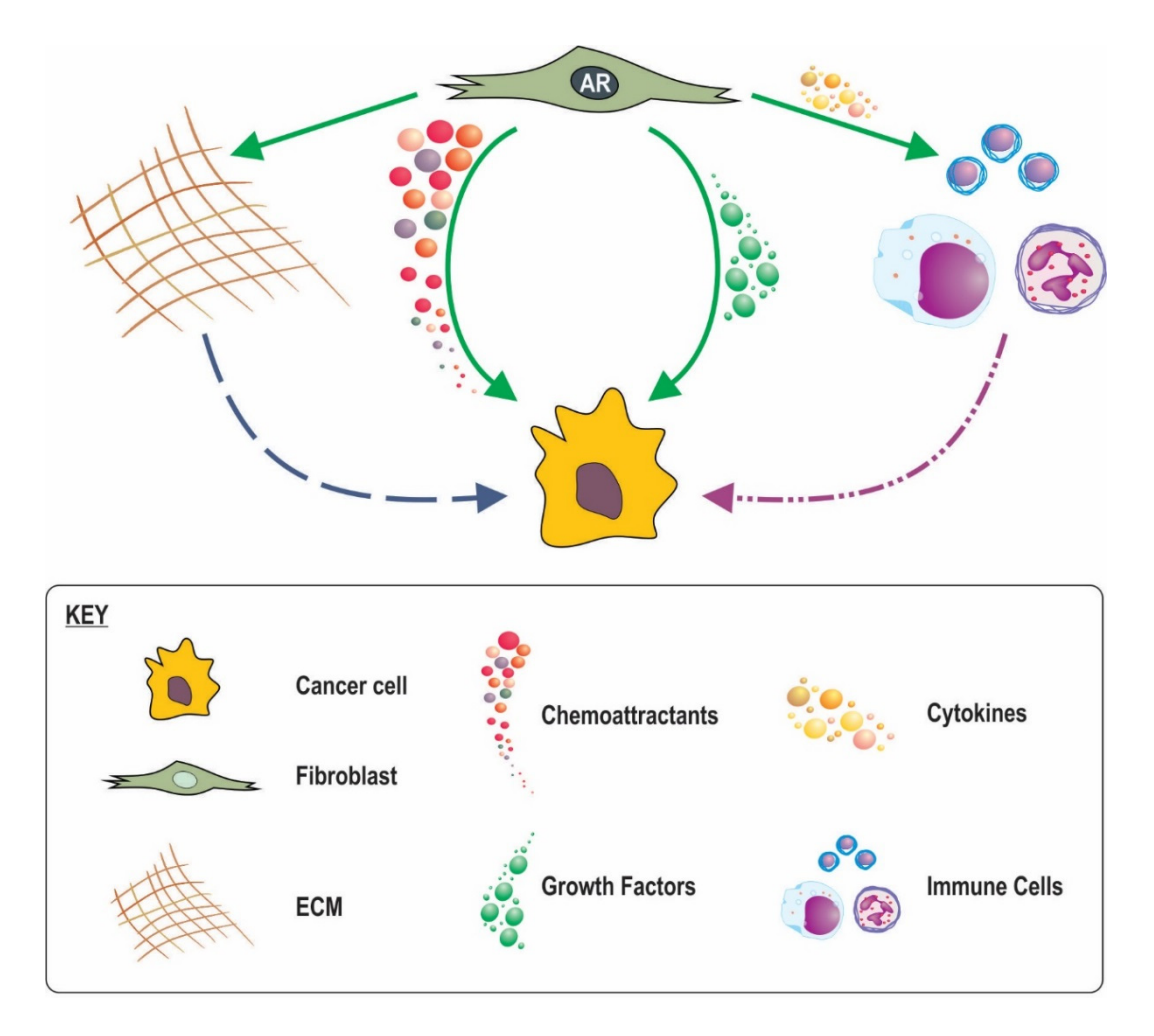
Potential mechanism for fibroblast AR influence on prostate cancer outcomes. AR signalling in fibroblasts regulates growth factors, chemoattractants, cytokines and ECM production. By regulating growth factors AR creates a hospitable environment for cancer, thus when AR is lost the local environment may drive cancer cells to metastasise elsewhere. AR regulates chemoattractant production, disruption of this may excite the migratory capacity of cancer cells. By regulating cytokine production, AR signalling in fibroblasts my influence immune response which may have significant effects on tumour cells. AR signalling in fibroblasts controls fibroblast production of ECM, when AR is lost, this could dysregulate the ECM and enhance the migratory potential of cancer by providing a transversable ECM microenvironment.

The mechanisms by which stromal AR action influences response of adjacent epithelia are slowly emerging. Secretion of factors by fibroblasts in response to androgens activate intracellular signalling pathways in epithelia as well as post translational modification of AR, increased AR activity [12,86], and stimulation of epithelial proliferation [87,88]. In contrast however, in transgenic adenocarcinoma of the mouse prostate (TRAMP) mice co-inoculated with AR negative highly metastatic human prostate cancer PC3 cells and human WMPY fibroblasts, knockdown of fibroblast AR with a specific siRNA did not alter cancer cell proliferation based on Ki67 index [89]. Reconciling the paradox between the apparent need for stromal AR signalling in the initial stages of cancer development versus the apparent importance of lost stromal AR signalling with cancer progression and outcome may have previously been problematic as there has been limited research into the function of AR in stromal cells. This dichotomy can now be recognized as not mutually exclusive as detailed below and surmised in Figure 3.

### 8.1. Loss of Stromal AR Creating Less Favourable Conditions

Fibroblasts produce a number of paracrine factors favourable for cancer cell growth (Table 3). A number of these paracrine factors are reported to be influential in cancer initiation and growth and their inhibition in fibroblasts is reported to alter cancer progression in vivo [90,91]. We and others have recently shown how fibroblast androgen action leads to regulation of a number of these paracrine factors in vitro, at least at an RNA level (Table) [12,87]. During prostate development moreover, androgen drives mesenchyme secretion of paracrine factors including FGFs, BMPs, WNTs, TGFBs and EPHs [92]. Furthermore stromal specific AR knockdown reduces mesenchymal production of key paracrine factors, IGF1, FGF7, FGF10, and HGF [56,71,93]. Indeed mouse models of androgen deprivation therapy (ADT) have reported marked reduction in stromal expression of FGF2 Il6, IGF1 and TGFB [91,94,95,96], all of which are capable of significantly increasing cancer cell proliferation and tumour growth [97], and acting to maintain terminal differentiation of the glandular epithelia [98]. An increased abundance of stimulatory growth factors by mesenchymal androgen action might thus contribute to the tumourigenic process. For initiated cancer cells however, decreased in local availability of paracrine mediators as the result of declining mesenchymal AR signalling could result in (i) de-differentiation and/or epithelial-mesenchymal transition (EMT); (ii) reduced epithelial AR function and PSA production that has implications for clinical monitoring via PSA and response to androgen deprivation therapy; and (iii) a less hospitable environment for epithelial cells thus driving pathways for epithelial movement and metastasis to more favourable sites.

### 8.2. A Role for Stromal in AR Inflammatory Processes

A high abundance of inflammatory cells is associated with development of prostate cancer and with poor outcome [101], and there is an association between age induced decline in testosterone and increased prostatic inflammation [102,103,104]. Although an anti-inflammatory effect of androgens has been demonstrated for the whole prostate [105], the role of fibroblasts, and indeed fibroblast AR signalling, in this process is unclear. Significantly however, fibroblasts are known to interact with inflammatory immune cells [106], and testosterone action in synovial fibroblasts has been suggested to have an anti-inflammatory role by inhibition of pro-inflammatory cytokine production [107,108]. Moreover, CAFs themselves have been reported to activate immune responses via NFKB secretion, while AR in prostatic fibroblasts is believed to modulate the release of pro-inflammatory cytokines that affect initiation and development of BPH and PIN [71]. The above data are collectively compelling for immune regulation in the prostate and a role in the tumour process, but the specific mechanisms and role of fibroblast AR need direct elucidation.

### 8.3. AR in CAF Movement and a Subsequent Role in Cancer Invasion

Compared to normal fibroblasts, CAFs have been shown to modulate movement of cancer cells through a variety of distinct mechanisms and effectors [90,109,110,111,112,113], and in themselves are more migratory than NPFs [114]. On one level, changes in fibroblast maintenance of ECM can serve to enhance movement of cancerous epithelia directly via independent matrix interactions [115,116]. On another, the ability of fibroblasts to move, create guidance structures, and dictate cancer cell movement may a key determinant in cancer progression and metastasis [115,117,118]. We have previously reported in fibroblasts a non-genomic role for AR and its co-regulator, Hic-5, in controlling fibroblast movement. With decreased androgen action, Hic-5 associates preferentially with the focal adhesion complex to inhibit its activity, facilitating fibroblasts detachment from the extracellular matrix and increased movement. It can be predicted therefore, that the loss of fibroblast AR might increase fibroblast movement and stimulate direct guidance of cancer cells. Furthermore, chemotactic cues are reported to outweigh any other conflicting stimuli, and drive migration [119]. Androgen also regulates the fibroblast expression of the potent chemo attractant, CXC12 [12,87]. The role of CXCL12 in controlling cancer cell movement is well known [120]. Additionally there are a host of other chemokines produced by CAFs which may similarly be regulated any androgen [56,88,121,122], and could provide an avenue by which disruption of AR signaling in fibroblasts may change the migrationary potential of cancer cells thus affecting patient outcomes.

### 8.4. Stromal AR Regulation of ECM

We recently hypothesised that the inverse relationship between stromal AR level and prostate cancer outcome is the result, in part, of changes in the production and regulation of fibroblast ECM [12]. The ECM is an intricate matrix of proteins and glycans that provide structural support for tissue and organs, and acts as a repository of hormones, enzymes and second messengers. It has been shown that the ECM can stimulate tumour growth and encourage cell cycle progression of cancer cells through proliferative checkpoints [123]. The ECM can also drive cancer cell gene expression, signal transduction, cell morphology, cell survival, and motility [124]. Changes in ECM can also cause CAFs to secret pro-inflammatory markers, thereby enhancing cancer progression [125,126]. In physical terms, it appears that the ECM can regulate cancer cell invasion via multiple parameters, including density, orientation, stiffness, and organisation of the matrix fibres. Whilst the effects of these different ECM characteristics can be interdependent or combine to create effects, it should be noted that they are independently able to affect cancer cell behaviour [127].

The role of ECM density is potentially complicated as well as controversial. Accompanying the switch from benign to malignant tissue for a number of different cancers is an increase in certain ECM proteins such as collagen 1. However, these reactive changes also coincide with a change from a mainly smooth muscle stroma that doesn’t produce much ECM, to one composed predominantly of high-ECM producing/maintaining fibroblasts and myofibroblast. These changes occur with all solid tumours, but nevertheless not every cancer will metastasise. In breast cancer, high collagen production is associated with cancer development and is reported to excite tumourigenesis and proliferation, and to alter intracellular processes to excite cancer cell movement [128,129,130]. While increased density may contribute to cancer initiation, it might conversely oppose tumour progression. As an example, hypoxia is a known driver of cancer progression and is associated with the ECM acquiring a loose and porous phenotype [131]. Although early 2-D ECM models suggested a relationship between density and cancer cell motility, more recent 3-D models show that cancer cells move more rapidly and easily through low density ECM [132,133,134]. The idea of androgen regulation is confirmed in vivo with a number of observations in ADT studies, noting changes in ECM volume [135,136,137,138] as well as changes in MMP levels [138,139]. Furthermore androgen regulates ECM component genes expression, and produces an ECM capable of altering cancer cell adhesion and migration [12].

The firmness or rigidity of the ECM fibres is also reported to affect cell movement. Traditionally, increased stiffness was believed to enhance migration by encouraging mesenchymal-type cell invasion [140], and by regulating cellular arrangement of integrins to control cell movement processes [129]. Conversely, increased stiffness and rigidity inhibits the ability of ECM fibres to be degraded by proteolytic enzymes such as MMP [141]. The recent move towards 3-D modelling has shed greater light on this process, specifically that maximal cell movement of cancer cells, such as human prostate DU145 cells, occurs in matrices exhibiting lesser stiffness [142].

Another aspect of the ECM that is accruing evidence for a major role in cancer progression is the orientation of the ECM fibres. In both in vitro and in vivo systems, cancer cells exhibit increased invasion and metastasis if ECM is arranged linearly to provide tunnels and tracts for cell movement. Similarly, the pore size, or space between ECM fibres can modulate cancer cell movement [132,134,143]. In in vitro 3D modelling, testing different poor sizes, widths, and arrangements, suggests that increased density and constricted poor sizes have an inhibitory effect on cell migration [140,144].

In summary, movement of cancer cells appears to be the culmination of intrinsic changes within the cell combined with the external influence and guidance of the ECM [145]. Fibroblasts AR has the ability to regulate the ECM, which when lost will create an environment favourable for cancer cell invasion and metastasis. This ability of AR signalling within fibroblasts to regulate the ECM may be key factor in stromal AR correlation with outcome and worthy of further investigation.

## 9. Potential Importance of Stromal AR in Neoadujant Hormone Therapy

As prostate cancers progress to hormone refractory metastatic disease, usually under conditions of androgen deprivation or complete androgen blockade, the epithelial AR is widely believed to have acquired the capacity to drive tumour growth. In early stage disease however, it appears as if the stromal AR is required in both tumour initiation and conversely as an inhibitor of progression and metastasis, and unlike its epithelial counterpart holds prognostic information. Additionally, in mouse recombinant models where patient cancer tissue is grown in the presence of either AR positive or negative fibroblasts, the apoptotic response of cancer cells to castration is significantly modulated by AR in the surrounding fibroblasts [12]. Given this dichotomy, we reviewed the use of ADT in a neoadjuvant setting for primary prostate cancer (Table 4). Despite ADT not usually deemed a standard treatment for organ confined prostate disease, the CaPSURE registry showed increasing trends since 1990 for the use of ADT in a neoadjuvant setting either alone or in conjunction with of other forms of treatment [146]. Neoadjuvant use of ADT does reduce primary tumour size by 25%–30% [147,148]. However, recent studies using pre-existing patient cohort information showed that neoadjuvant ADT as a front-line therapy led to greater relative mortality when compared to surgery or radiation in a cohort of 7538 prostate cancer patients [149]. In a second population-based study of over 1900 men with T1–T2 prostate cancer, the use of ADT as primary therapy was associated with a lower rate of prostate cancer-specific survival [150]. In a study of 16,000 men with well-to-moderately differentiated tumours, the use of primary ADT within the first six months of diagnosis was associated with worse rates of overall survival and prostate cancer specific mortality, regardless of any additional treatment after this first 6 months [151]. A similar finding was reported by the European Organization for Research and Treatment of Cancer (EORTC) clinical trial, which investigated immediate and delayed use of ADT for treatment of locally defined tumours [152]. The use of ADT for localized prostate cancer increased the subsequent need for chemotherapy [153]. Nonetheless, there have been other reports suggesting either no or a slight beneficial effect of primary ADT [154,155], but these have had significantly smaller cohorts of 176 and 57 patients, respectively. Likewise, in a larger study of 1006 patients with low to intermediate prostate cancer treated with low dose brachytherapy (LDB), the use of ADT either three months prior to or concomitantly with LDB did not affect disease free or overall patient survival [156]. Furthermore, studies that have reported unconventional forms of primary ADT (i.e., diethylstilbestrol) have had inconsistent results with benefit for T2 tumours but deleterious effects for T1 disease [157]. Overall, the evidence suggests that neoadjuvant use of ADT may produce harmful effects through unknown mechanisms. However as discussed above, ADT is of well proven benefit in metastatic disease so the adverse response of this treatment when used in a primary setting must be due to adverse targeting/response of the early stage tumours. It is entirely possible that this paradox is due to effects of androgen signalling in cancer fibroblasts associating with the primary/early stage lesions.

## 10. Future of Stromal AR

### 10.1. Prognostic Tool

There is growing appreciation for the influence of stroma in cancer, so much so that a number of studies have looked to the stroma for prognostic utilisation. Morphological characterisation of prostate cancer has used the degree of desmoplatic stroma to predict biochemical recurrence and cancer related death [169,170,171]. Stromal signatures and protein profiles have been investigated, and have been used to predict relapse post prostatectomy and clinical outcome [77,172,173,174]. Clinically, no protein expression or gene profiles are used to aid prognosis, despite the various immunohistochemical markers used in other cancers, such as breast cancer where oestrogen and progesterone receptors are used to inform on disease coarse and management. Along these lines we, and others have studied the benefit of using stromal AR in clinical settings. Despite inconsistent findings for the prognostic values for epithelial AR, a loss of stromal AR is consistently associated with disease relapse and outcome [7,8,9,10,11,12,41] (Table 2). We have also found using FKBP51 in addition to AR, as a marker of functional AR activity is even more robust prognostic tool [13]. These studies have focus on tissue samples, development of serum markers for stromal AR changes may also be useful tool. From whole genome studies we know a number of genes targeted by AR fibroblasts code for secreted proteins so with further work there may be potential for development of serum markers.

### 10.2. Therapeutic Targets

Just like in the prognostic setting, the cancer stroma is being investigated for its therapeutic influence and even as a target. The important role of CAFs have led to monoclonal antibodies and drugs which target the CAF marker, fibroblast activated protein (FAP) [175,176,177]. The stroma surrounding the tumour is exposed to any serum administered therapeutic agent before said therapeutic agent reacts with the cancer. Indeed it has been postulated that the stroma will mediate the influence of the therapeutic agent [178].

Therapeutic antibodies and small molecule inhibitors delivered in nanoparticles as well as extracts from natural compounds are being investigated for disrupting paracrine communication between the stroma and cancer cells to treat solid cancers [179,180]. A number of stromal produced paracrine factors, regulated by AR have been targeted therapeutically to varying degrees of success. Androgen regulated paracrine factors such as TGFs, FGFs, EGF, HGFs produced by the stroma having agents capable of targeting them [178]. FGF targeting has been reported to be effective in both in vitro and in vivo studies for treating prostate cancer [181,182]. Similarly, agents targeting HGF in prostate cancer are in different phases of clinical trial [183,184].

However, no therapeutic agents have been developed to specifically target stromal AR. Indeed in cases of neoaduvent ADT or use of AR antagonists the effect on stromal AR and the subsequent effects of stromal AR inhibition is rarely considered. In review of studies investigating the use of ADT on primary prostate tumors, the neoadjuvant use of ADT predominantly produces worse outcomes for the patients, with relapse free survival and overall survival reduced. Given the relationship between reduced stromal AR and cancer related progression and death, it may be more important to investigate either anti-androgen which affect only epithelial cells, or developing drugs which will decrease epithelial AR but enrich stromal AR signalling. As we have previously shown a single co-regulator can have vast effects on global gene expression with the cell. One way to ensure specificity would be to target AR co-regulators and pioneer factors, a number of which are specific for one cell type or the other [46]. In comparison of prostatic and skin fibroblasts to cancer cell lines, a panel of 33 co-regulators were differentially expressed between the two cell types [46]. Cancer cell type specific co-regulators included SP1, NCOA1, NCOA2, and PIAS1. Importantly these are potentially targetable [185,186]. Pioneer factors are also targetable, and as we have shown FOXA1 is expressed and active only in epithelial cells and not fibroblasts [43,186]. However targeting Hic-5, AP-1, or other proteins which is also or highly expressed in the stroma should be avoided as inhibiting stromal AR may have detrimental side-effects. Taking into account stromal AR should become an important step in future development of treatments targeting AR signalling, especially in a neoadjuvent setting.

## Figures and Tables

**Table 1:** Expression of AR in cancerous epithelial tissue and association with outcomes. RP = Radical prostatectomy; TURP = Transurethral resection of the prostate; IHC = Immunohistochemistry; RT-PCR = Real time polymerase chain reaction.

Authors	Specimens	Cohort Size	Methods	Effect on Prostate Cancer Outcome
[14]	Biopsies	62	IHC	Higher AR, better prognosis
[15]	Biopsy, RP and TURP	42	IHC	Higher AR, better prognosis
[16]	Biopsies	90	IHC	Higher AR, better prognosis
[17]	RP	197	IHC	Higher AR, better prognosis
[18]	RP	105	IHC	Higher AR, better prognosis
[19]	mixed RP, TURP, Biopsy	42	IHC	Higher AR, better prognosis
[9]	RP	96	IHC	Higher AR, biochemical relapse
[20]	RP	115	RT-PCR	Higher AR, biochemical relapse
[21]	RP	340	IHC	Higher AR, biochemical relapse
[22]	RP	52	IHC	Higher AR, biochemical relapse
[8]	RP	53	IHC	Higher AR, biochemical relapse
[22]	RP	52	IHC	Higher AR, worse prognosis
[23]	RP	640	IHC	Higher AR, worse prognosis
[24]	mixed RP/biopsy	66	IF	Higher AR, worse prognosis
[11]	RP	56	IHC	Not prognostic
[25]	RP	232	IHC	Not prognostic
[26]	TURP	68	IHC	Not prognostic
[27]	RP	64	IHC	Not prognostic
[28]	Biopsies	17	IHC	Not prognostic
[29]	RP	121	RT-PCR	Not prognostic
[30]	TURP and RP	81	IHC	Not prognostic
[31]	RP and metastases	119	IHC	Not prognostic
[32]	RP	2805	IHC and RT-PCR	Not prognostic
[33]	RP	172	IHC	Not prognostic
[34]	TURP	24	IHC	Not prognostic
[10]	TURP + biopsy	154	IHC	Not prognostic
[35]	RP	43	IHC	Not prognostic
[7]	RP	20	IHC	Not prognostic
[12]	TURP	64	IHC	Not prognostic
[36]	RP	53	branched chain DNA	Not prognostic
[37]	RP	10	IHC	Unavailable
[38]	Biopsies	39	IHC	Unavailable
[39]	RP	26	IHC	Unavailable
[40]	RP	50	IHC	Unavailable

**Table 2:** Expression of AR in cancerous stroma and association with patient outcomes. RP = Radical prostatectomy; TURP = Transurethral resection of the prostate; IHC = Immunohistochemistry.

Authors	Specimens	Cohort Size	Methods	Effect on Prostate Cancer Outcome
[41]	RP	44	IHC	Low AR, biochemical relapse
[8]	RP	53	IHC	Low AR, biochemical relapse
[9]	RP	96	IHC	Low AR, biochemical relapse
[12]	TURP	64	IHC	Low AR, PCSM
[10]	TURP + biopsy	152	IHC	Low AR, worse prognosis
[11]	RP	56	IHC	Low AR, worse prognosis
[7]	RP	20	IHC	(low AR, no association with Gleason)

**Table 3:** Stromal produced paracrine factors. Proliferative effect (P), Differential effect (D) supported by [97,99,100]. Androgen regulation (Y = yes, regulated by androgen, N = no, not regulated by androgen) determined from microarray data from [12,45,87].

Paracrine Factor	Effect	Androgen Regulation
CTGF	P	Y
FGF (2, 5, 7, 8, 9, 10)	P, D	Y (2, 5, 7), N (8), *N*/*A* (9, 10)
HGF	P, D	Y
IGF (1, 2)	P, D	Y (1, 2)
IL-6	P	Y
PDGF	P, D	Y
TGFb (1, 2, 3)	P, D	Y (1, 2, 3)
VEGF (A, B, C)	P	Y (A,C), N (B)
WNT	P	Y
CXCL12	P	N
EGF	P, D	*N/A*
TGFa	P, D	*N/A*

**Table 4:** Outcomes from studies investigating effects of neoadjuvant ADT and outcomes of patients with localized prostate cancer.

References	N	Pca Staging	ADT Use	Comparison	Outcome
[149]	7538	T1–T3	Neo	ADT vs. surgery or radiation	ADT increases hazard ratio
[150]	19,271	T1–T2	Neo (<180 days)	ADT vs. conservative management	Decreased PCSS
[151]	16,000	T1–T2	Neo (<first 6 months)	ADT in first 6months vs. no ADT in first 6 months	Increased PCSM
[153]	29,775	Localized	Neo	ADT vs. noADT	ADT increases need for subsequent treatments
[158]	844		Neo (<first 6 months)	Neo compared to WW, RP, radiotherapy	Neo had worse 10 year PCSS of all treatments
[159]	10,179	Localised	Neo	Neo compared to no treatment, RP, BT, ERBT	ADT worse PCSS
[160]	402	Localised	Neo (<first 3 months)	Neo compared to RP alone	Neo = pathological downstaging and lowers % of patients with positive margins
[161]	547	Localised	Neo	3-month vs. 8-month neo	Positive margin rates were significantly lower in the 8 than 3-month group
[162]	167	T1a–T2b	Neo (<first 3 months)	3-month neo vs. RP alone	Neo had less lymph node involvement, less positive margins
[163]	393	T2–T3	Neo (3–6 months)	Neo vs. RP alone	Neo had better positive margin rates
[164]	119	T2–T3a	Neo (<first 4 months)	4-month neo vs. RP alone	Neo had better positive margin rates
[154]	176	B2/T2–T3	Neo	1-year ADT vs. long term ADT	No measurable significant benefit
[155]	57		Neo		No benefit
[156]	1006	Low-intermediate	Neo ADT + LDB	ADT prior to or after LDB	No effect of PCSS
[165]	282	T2b	Neo (<first 3 months)	3-month neo vs. RP alone	No difference in 5 year BCR
[166]	126	T1b–T3aNXM0	Neo (<first 3 months)	3-month neo vs. RP alone	No difference in PSA progression-free survival (7 year follow up)
[167]	148	T1b–T3	Neo (<first 3 months)	3-month neo vs. RP alone	No significant difference in BCR-free (8 year followup)
[152]	985	Localized	ADT Immediately or upon symptoms of progression	Immediate ADT vs. delayed ADT	Delayed ADT increased risk of mortality
[157]	1903	T1–T2	Neo (diethylstilbesterol)	ADT in T1 vs. ADT in T2	benefit T2, deletrious in T1
[168]	213	T1b/c–T2c	Neo	Neo prior to surgery vs. surgery alone	Neo = less organ confinement, lower 7-year survival
